# New Books

**Published:** 2008-12

**Advances in Bioactivation Research**

Adnan A. Elfarra, ed.

New York:Springer, 2009. 504 pp. ISBN: 978-0-387-77299-8, $189

**Bioinformatics and Functional Genomics, 2nd ed.**

Jonathan Pevsner

Hoboken, NJ:John Wiley & Sons, Inc., 2009. 897 pp. ISBN: 978-0-470-08585-1, $225

**Bringing the Sun Down to Earth: Designing Inexpensive Instruments for Monitoring the Atmosphere**

David R. Brooks

New York:Springer, 2008. 150 pp. ISBN: 978-1-4020-8693-9, $79.95

**Changes in the Human-Monsoon System of East Asia in the Context of Global Change**

Congbin Fu, J.R. Freney, J.W.B. Stewart, eds.

Hackensack, NJ:World Scientific, 2008. 384 pp. ISBN: 978-981-283-241-2, $88

**Climate Change and Technological Options**

Konrad Soyez, Hartmut Graßl

New York:Springer, 2008. 219 pp. ISBN: 978-3-211-78202-6, $139

**Climatic Changes and Water Resources in the Middle East and North Africa**

F. Zereini, H. Hötzl, eds.

New York:Springer, 2008. 552 pp. ISBN: 978-3-540-85046-5, $229

**Ecological Impacts of Climate Change**

Commitee on Ecological Impacts of Climate Change, National Research Council

Washington, DC:National Academies Press, 2008. 70 pp. ISBN: 978-0-309-12710-3, $18.90

**Emerging Environmental Technologies**

Vishal Shah, ed.

New York:Springer, 2008. 174 pp. ISBN: 978-1-4020-8785-1, $109

**Encyclopedia of Genetics, Genomics, Proteomics, and Informatics**

George P. Rédei

New York:Springer, 2008. 2,202 pp. ISBN: 978-1-4020-6753-2, $999

**Global Climate Change and Extreme Weather Events: Understanding the Contributions to Infectious Disease Emergence: Workshop Summary**

David A. Relman, Margaret A. Hamburg, Eileen R. Choffnes, Alison Mack

Washington, DC:National Academies Press, 2008. 304 pp. ISBN: 978-0-309-12402-7, $62.25

**Hijacking Sustainability**

Adrian Parr

Cambridge, MA:MIT Press, 2009. 224 pp. ISBN: 978-0-262-01306-2, $24.95

**Information Resources in Toxicology, 4th ed.**

Philip Wexler, P.J. Hakkinen, Asish Mohapatra, Steven G. Gilbert

St. Louis, MO:Elsevier, 2009. 1,296 pp. ISBN: 978-0-12-373593-5, $199.95

**Pharmaceuticals in the Environment**

Klaus Kümmerer, ed.

New York:Springer, 2008. 522 pp. ISBN: 978-3-540-74663-8, $189

**Physics of the Environment**

A.W. Brinkman

Hackensack, NJ:World Scientific, 2008. 228 pp. ISBN: 978-1-84816-179-5, $75

**Principles and Practice of Skin Toxicology**

Robert Chilcott, Shirley Price, eds.

Hoboken, NJ: John Wiley & Sons, Inc., 2008. 392 pp. ISBN: 978-0-470-51172-5, $140

**Safe Use of Chemicals: A Practical Guide**

T.S.S. Dikshith

Boca Raton, FL:Taylor and Francis Group, 2008. 312 pp. ISBN: 978-1-420-08051-3, $82.93

**Science and the Media**

Peter Snyder, Linda Mayes, Dennis Spencer

St. Louis, MO:Elsevier, 2008. 272 pp. ISBN: 978-0-12-373679-6, $49.95

**The Yearbook of Nanotechnology in Society. Vol. 1: Presenting Futures**

Erik Fisher, Cynthia Selin, Jameson M. Wetmore, eds.

New York:Springer, 2008. 310 pp. ISBN: 978-1-4020-8415-7, $149

